# RELATIONSHIP-TYPE DIFFERENCES IN SUPPORT SEEKING AND MENTAL HEALTH FOR ADULT CHILDREN OF PARENTS WITH MEMORY LOSS

**DOI:** 10.1093/geroni/igad104.2554

**Published:** 2023-12-21

**Authors:** Dustin Gad, Joan Monin

**Affiliations:** Yale School of Public Health, New Haven, Connecticut, United States; Yale School of Public Health, New Haven, Connecticut, United States

## Abstract

Research shows that midlife children still rely on their parents for emotional and tangible support, which has implications for their mental health. Little is known about how these dynamics change when a parent enters the early stage of dementia or memory loss. One characteristic that may be important for these dynamics is the relationship-type (daughter-mother (n= 82), daughter-father (n= 29), son-mother (n=17), son-father (n= 14)). As part of a larger study, adult children 18 years of age and older (n= 142) completed self-report surveys including measures of emotional and tangible support seeking from their parent (Feeney, 2004), the 10-item CESD (Orme et al., 1986), the 12-item Zarit Burden Inventory (Bédard et al., 2001), and the PANAS (Watson et al., 1988). Results showed that tangible and emotional support seeking levels were low across all dyad-types, with no differences between groups. There was a trend for depressive symptoms (F= 2.559, p= .058) showing more in daughter-father than in son-mother dyads. Daughter-father dyads were the only group meeting the depression cut-off score. There were significant differences for burden (F= 3.377, *p= .020) with more burden in daughter-mother compared to son-father dyads. For the PANAS (F= 2.926, *p= .036), there was more negative affect in daughter-father compared to daughter-mother dyads. Results suggest that the relationship-type of the adult child and parent matters for mental health in the early stages of memory loss. This has implications for dyadic interventions helping adult children and their parents cope in the early stages of the dementia caregiving journey.

